# Robust Hippocampal Synaptic Plasticity Despite Gut Microbiota Depletion in Adult Mice

**DOI:** 10.1111/ejn.70346

**Published:** 2025-12-18

**Authors:** Michael K. Collins, Henry Darch, Loreto Olavarría‐Ramírez, Cian McCafferty, Kenneth J. O'Riordan, John F. Cryan

**Affiliations:** ^1^ APC Microbiome Ireland University College Cork Cork Ireland; ^2^ Department of Anatomy and Neuroscience University College Cork Cork Ireland

## Abstract

The microbiota‐gut‐brain axis describes the bidirectional communication between the brain and the trillions of microorganisms living in the gut. Moreover, current evidence suggests that this axis can influence host behaviour and brain physiology. Previously we have shown that adult mice that have not been exposed to microbes throughout their lives display sex‐specific deficits in hippocampal synaptic plasticity. However, it is not known whether this phenomenon originates during neurodevelopment or whether similar effects could be recreated with microbiome depletion in adulthood. Therefore, we explored the vulnerability of hippocampal synaptic function to altered microbiome signals, depleting the microbiome of male and female mice for 2 weeks with either an antibiotic cocktail or a single antibiotic added to drinking water. The antibiotic cocktail contained a variety of antibiotics including broad‐spectrum antibiotics to ensure widescale microbiota depletion (ampicillin, vancomycin and imipenem). In addition, a more targeted depletion of Gram‐positive gut bacteria was conducted using the gut‐restricted antibiotic vancomycin. Ex vivo hippocampal electrophysiology measures of basal synaptic efficacy, short‐term plasticity, and long‐term potentiation (LTP) were then examined. We found that there was no effect of antibiotic administration on any of these measures, demonstrating the robustness of these hippocampal circuits to microbiome depletion during early adulthood. Taken together, this shows the ability of adult hippocampal plasticity to withstand a gut microbiome insult.

## Graphical abstract

**Microbiota depletion in adulthood does not alter hippocampal synaptic function in male and female mice.** We investigated whether short‐term depletion of the gut microbiota in adulthood alters hippocampal synaptic physiology. Neither broad‐spectrum nor Gram‐positive‐targeted antibiotics impaired hippocampal synaptic plasticity in male and female adult mice, suggesting adult hippocampal plasticity is able to withstand a gut microbiome insult.

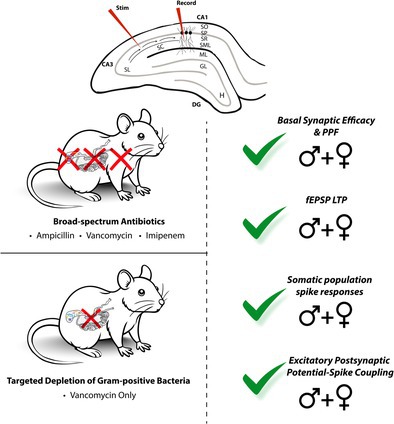

**Abbreviations**
ABX: antibiotic cocktail
aCSF: artificial cerebrospinal fluid
CA1: cornu ammonis area 1
CaCl_2_
: calcium chloride
CO_2_
: carbon dioxide
Ctrl: control mice
DG: dentate gyrus
E‐S: EPSP‐spike
fEPSP: field excitatory postsynaptic potential
GF: germ‐free
GL: granule cell layer
H: hilus of the dentate gyrus
I/O: input/output
IPI: interpulse interval
KCl: potassium chloride
LTP: long‐term potentiation
MEA: multielectrode array
MgSO_4_
: magnesium sulphate
ML: molecular layer of the dentate gyrus
NaCl: sodium chloride
NaH_2_PO_4_
: sodium dihydrogen phosphate
NOR: novel object recognition
PPF: paired‐pulse facilitation
SL: stratum lucidum
SML: stratum lacunosum‐moleculare
SO: stratum oriens
SP: stratum pyramidale
SR: stratum radiatum
TBS: theta burst stimulation

## 1 Introduction

Emerging evidence indicates the gut microbiome is crucial to host behaviour and disease modulation (Cryan et al. 2019). Among influenced behaviours and processes are cognitive functions including learning and memory (Kuijer and Steenbergen 2023). Notably, gut microbial composition is correlated with hippocampal function, a key brain region involved with learning and memory (Alemohammad et al. 2022; Renson et al. 2020). Furthermore, significant gut microbiota composition differences exist between healthy individuals and Alzheimer's disease patients, suggesting a potential role of the gut microbiome in cognition and memory (Chandra et al. 2023).

Synaptic plasticity, a major cellular mechanism underlying learning and memory (Kennedy 2013), is an activity‐dependent process involving enduring changes to synaptic strength (Bliss and Lomo 1973). The microbiome has a significant capacity to affect this process in the hippocampus. Probiotic administration may mitigate age‐related deficits in long‐term potentiation (LTP), a form of synaptic plasticity (Distrutti et al. 2014). Additionally, gut microbiome transplantation from young to old mice has been found to attenuate age‐related deficits in cognitive behaviours alongside hippocampal transcriptome changes (Boehme et al. 2021). Finally, we previously discovered a sex‐dependent LTP deficit in germ‐free (GF) mice, which were raised in an environment devoid of microbial influence (Darch et al. 2021). It remains unclear whether this deficit is due to neurodevelopmental alterations from their GF upbringing, or whether continuous microbial signalling is necessary to maintain normal hippocampal synaptic plasticity.

Antibiotic exposure can impact learning and memory, but studies often examine only one sex. Previous research shows antibiotic treatment decreases memory retention in female mice (Mohle et al. 2016), and in male mice disrupts novel object recognition (NOR) but not spatial memory (Frohlich et al. 2016). Other studies provide evidence supporting the synaptic‐plasticity–altering potential of antibiotics (Bercik et al. 2011; Çalışkan et al. 2022; Cordella et al. 2021). Further, GF or antibiotic‐exposed male mice microbiome perturbation shows increased hippocampal BDNF, a factor strongly associated with synaptic plasticity (Lu et al. 2014; Neufeld et al. 2011).

Given sex‐dependent deficits in synaptic plasticity in GF mice and the impact of antibiotics on learning and memory in adulthood, we pose the following question: Can sex‐dependent deficits in synaptic plasticity be replicated through microbiome depletion in adulthood? To address this question, we depleted the microbiome of adult male and female mice using two antibiotic regimens and subsequently performed ex vivo hippocampal electrophysiology.

## 2 Methods

### 2.1 Animals

Adult male and female C57BL/6 mice (Male N Control/Antibiotic Cocktail/Vancomycin = 11/9/9, Female Control/Antibiotic Cocktail/Vancomycin = 7/7/6; 8–10 weeks; ENVIGO, UK) were used. All experiments were conducted in accordance with the European Communities Council Directive 2010/63/EC, the requirements of the S.I. No. 543 of 2012 and approved by the Animal Experimentation Ethics Committee of University College Cork and the Health Products Regulatory Authority (HPRA AE19160/P118).

Animals were habituated in the animal facility for at least 1 week before experiments started and kept under a 12‐h light/dark cycle, at 21°C ± 1°C with a humidity of 55% ± 10%. Food and water were provided ad libitum.

### 2.2 Study Design

Gut microbiota from experimental animals was depleted using one of two approaches. First approach: a wide‐spectrum antibiotic cocktail (ABX) consisting of ampicillin (1 g/L, CAS no. 69‐52‐3), vancomycin (0.5 g/L, CAS no. 1404‐93‐9) and imipenem (0.25 g/L, CAS no. 74431‐23‐5). Second approach: a single antibiotic, vancomycin (0.5 g/L, CAS no. 1404‐93‐9), which targets Gram‐positive bacteria. Both treatments were prepared freshly with tap water every second day for 2 weeks (Frohlich et al. 2016). Control mice (Ctrl) received tap water. Water consumption in experimental groups (ABX and vancomycin) was recorded to ensure animals were drinking the antibiotics. Each cage housed two or three mice.

Cages were assigned to treatment groups sequentially along the cage rack (e.g., cage 1 = control, cage 2 = antibiotic cocktail, cage 3 = vancomycin). While this approach was not based on a formal randomisation algorithm, it avoided experimenter selection bias and ensured that an equal proportion of each treatment group was exposed to similar environmental conditions (e.g., light exposure and airflow) along the rack. Experimental recordings were scheduled in a quasi‐random sequence, with treatment groups alternated approximately every 2 days to minimise potential bias from day‐to‐day variation in electrophysiology performance.

We acknowledge that blinding was not implemented throughout. Treatment identity could be inferred during animal handling (e.g., from treatment bottles) and during dissection, as the caecum appearance differed markedly between control and antibiotic‐treated animals. While this could not be fully mitigated in the current study, this limitation is now noted explicitly in Section 4.

After 2 weeks of treatment, the mice were culled, and the hippocampi were removed for electrophysiological examination.

### 2.3 Artificial Cerebrospinal Fluid Content

Artificial cerebrospinal fluid (aCSF) was made up of (in mM): NaCl (124), KCl (2.7), NaH_2_PO_4_ (1.25), CaCl_2_ (2), MgSO_4_ (1.3), D‐Glucose (18) and ascorbic acid (2). Cutting aCSF was made up of the same aCSF, with excess MgSO_4_ (8.3 mM) and cooled to between 0°C and 4°C.

### 2.4 Slice Preparation

Hippocampal slice electrophysiology is a well used technique and our methods followed common practices (Papouin and Haydon 2018) and our previous publication (Darch et al. 2021). Fresh aCSF was made and bubbled with carbogen (95% O_2_/5% CO_2_) for at least 40 min prior to use and continually throughout recordings.

To minimise differential stress responses, all animals were left to acclimate to the experimental room for at least 1 h following transfer from the animal unit. Ice‐cold cutting aCSF (see recipe above) was used throughout dissection. Mice were rapidly decapitated, and the brain was removed and cooled with aCSF. The caudal third of the brain was removed with a razor blade, making a cut just rostral to the cerebellum. This created a flat brain surface to be glued to the stage of a Leica VT1200 vibratome. An agar block support was also glued on the stage against the brain's ventral side to aid slicing stability. 300‐μm coronal slices of the brain were cut.

Slices were transferred to a chilled Petri dish, filled with oxygenating aCSF, and the hippocampus was subdissected from the slices. A selection of slices spanning the central portion of the dorsoventral axis (approximately 1.5–2.1 mm from the dorsal end) was transferred to mesh holding wells in a bath of standard aCSF held at 32°C for 45 min and then at room temperature for 45 min.

After this recovery period, slices were transferred to a multielectrode array (MEA) chip (Multichannel Systems, Germany) and perfused with aCSF at 31°C–32°C. Slices were viewed under an inverted microscope (4× magnification, Olympus IX70) and compared for structural integrity and general slice health. The best candidate slice was chosen based on clearly observable *stratum pyramidale* and the best initial response to test stimulation. This slice was therein used for electrophysiological testing.

### 2.5 Electrophysiology Recording

**FIGURE 1:**
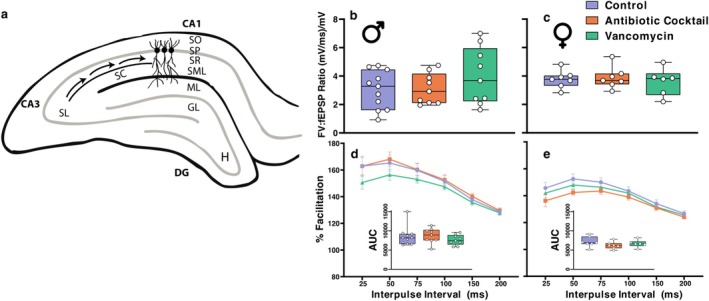
(a) Diagram of hippocampal slice featuring hippocampal layers and Schaffer Collateral (SC) pathway from CA3 to CA1. DG, dentate gyrus; GL, granule cell layer; H, hilus of the dentate gyrus; ML, molecular layer of the dentate gyrus; SL, stratum lucidum; SML, stratum lacunosum‐moleculare; SO, stratum oriens; SP, stratum pyramidale; SR, stratum radiatum. (b,c) Basal excitability summarised with the linear slope of each slice through an ‘input–output’ protocol measuring responses through a series of increasing stimulation strengths. (d,e) Paired‐pulse facilitation of fEPSPs. No statistically significant differences were found between slices pre‐ and post‐SCFA treatment. Error bars represent standard error of the mean. Male *N* Control/Antibiotic Cocktail/Vancomycin = 11/9/9, Female Control/Antibiotic Cocktail/Vancomycin = 7/7/6.

The chosen slice was placed onto an MEA chip (Menigoz et al. 2016; Shaban et al. 2017) and aligned so that a stimulating electrode was located over the Schaffer collateral pathway, and a recording electrode was situated in the middle of the CA1 *stratum radiatum* (200 μm ‘downstream’ of the stimulus electrode) (O'Dell et al. 1991). The slice was immobilised with a nylon mesh slice anchor. The MEA chip allowed for an additional recording electrode to be located in the *stratum oriens*, to record the somatic *pop*ulation spike of the stimulated neurons (Figure 1a). aCSF (heated to 31°C–32°C) perfusion rates were 2.5 mL/min in and 4 mL/min out, which optimally maintained fluid volume while minimising turbulent flow forces.

After testing the presence of field excitatory postsynaptic potential (fEPSP) responses with a small bipolar current (20 μA), the slice was left to recover with bipolar test pulse stimulation every 5 min to monitor recovery.

Once fEPSP slope measurements were stable (±15%) for approximately 15 min (typically 1.5 h after placement), an input/output (I/O) curve was computed by increasing stimulation current from 1 to 150 μA with a pulse every 30 s and measuring the slopes of the resultant fEPSPs, taking an average of three sequential stimulation ramps. All subsequent stimulation was performed at an intensity eliciting approximately 35% ± 5% of the maximum response of each slice. Subsequently, short‐term synaptic plasticity was tested with a paired‐pulse facilitation (PPF) protocol. Interpulse intervals (IPI) used were 25, 50, 75, 100, 150 and 200 ms. A 30‐s interval between each of these paired pulses was maintained, and this series of six pairs was repeated three times.

Following I/O and PPF tests, the slice was again stimulated using single‐bipolar pulses every 30 s. To test LTP, a baseline consisting of 30 min of stable recordings was acquired, during which the fEPSP slope did not exceed ±10% of itself for more than 2 min. Immediately following this baseline, the pathway was stimulated with three theta burst stimulations (3×TBS) to induce LTP. Each TBS consisted of four 100‐Hz pulses, repeated 10 times at 5 Hz, with an interburst interval of 30 s. After LTP induction, the stimulation frequency reverted to a single pulse every 30 s as with baseline for 2‐h post‐tetanus.

Stimulation protocols were designed in MCStimulusII (Multichannel Systems, Germany) and played during recordings via MCRack (Multichannel Systems, Germany).

### 2.6 Data Analysis and Statistical Methods

Data was converted to ‘.abf’ files and the slope (capturing 80%–90% of the inward current slope) of fEPSPs was extracted with Clampfit software. To compute population spike amplitude, we subtracted the trough of the spike from an average of the two adjacent peaks. For I/O relationships and PPF, the average of three responses at each stimulus intensity/interval was used. In testing LTP, data values of fEPSP slopes were expressed as a percentage of the average of the 30‐min baseline period fEPSPs, and an average of the final 10 min of this baseline (20 stimuli) was used for statistical analysis and representation in LTP bar chart figures.

Parametric tests were utilised for statistical analyses after appropriate data normality checks. Specific test details are given in the results. Individual slices (each from an individual animal) were treated as subjects; sex and treatment group status of animals were treated as fixed factors. Data originating from individual slices were treated as repeated measures (post‐TBS data).

## 3 Results

### 3.1 Microbiome Depletion Does Not Affect Basal Synaptic Efficacy or Short‐Term Plasticity in Adult Mice

To test whether microbiome depletion affected basal synaptic efficacy in hippocampal slices, we measured the fibre volley and resulting fEPSP at a variety of stimulus intensities of the I/O test. We summarised each slice's data through calculating the linear slope of this relationship (Kim et al. 2005; Woo et al. 2005) (Figure 1b,c). A univariate general linear model revealed that neither microbiome depletion with an antibiotic cocktail nor vancomycin on its own affected basal synaptic efficacy (*F*(2, 43) = 0.6, *p* = 0.692). No effect of sex was found (*F*(1, 43) = 0.6, *p* = 0.330). This suggests microbiome depletion does not affect basal synaptic excitability despite male vancomycin‐treated slices exhibiting a tendency toward increased values (Figure 1b).

In conventional animals under normal conditions, when two excitatory stimuli occur within a short space of time, the second postsynaptic response will be enhanced (Creager et al. 1980). This facilitation results from presynaptic mechanisms increasing neurotransmitter release (Jackman and Regehr 2017). To establish whether microbiome depletion affected presynaptic function, we tested short‐term plasticity using PPF. Maximal facilitation reached approximately 170% in male slices and 150% in female slices at a 50 ms IPI. Linear mixed model analysis revealed no main effect of microbiome depletion at any IPI. However, the greatest effect size difference was seen between male Ctrl and Vancomycin‐treated groups at the 25 ms IPI (Figure 1d). A main effect of sex was found at 200 ms (*F*(1, 43) = 11.185, *p* = 0.002). Pairwise comparisons revealed this effect was only present between males and females of ABX‐treated groups (*F*(1, 43) = 9.236, *p* = 0.004). While no interaction effect was present between sex and treatment (*F*(1, 43) = 0.964, *p* = 0.389), the lack of a sex difference between Ctrl and Vancomycin‐treated groups, while one exists in ABX‐treated groups, does indicate a subtle sex‐dependent effect of a more restricted microbiome depletion as opposed to a broad knockdown of the microbiota.

### 3.2 Microbiome Depletion Leaves Hippocampal LTP Unchanged

**FIGURE 2:**
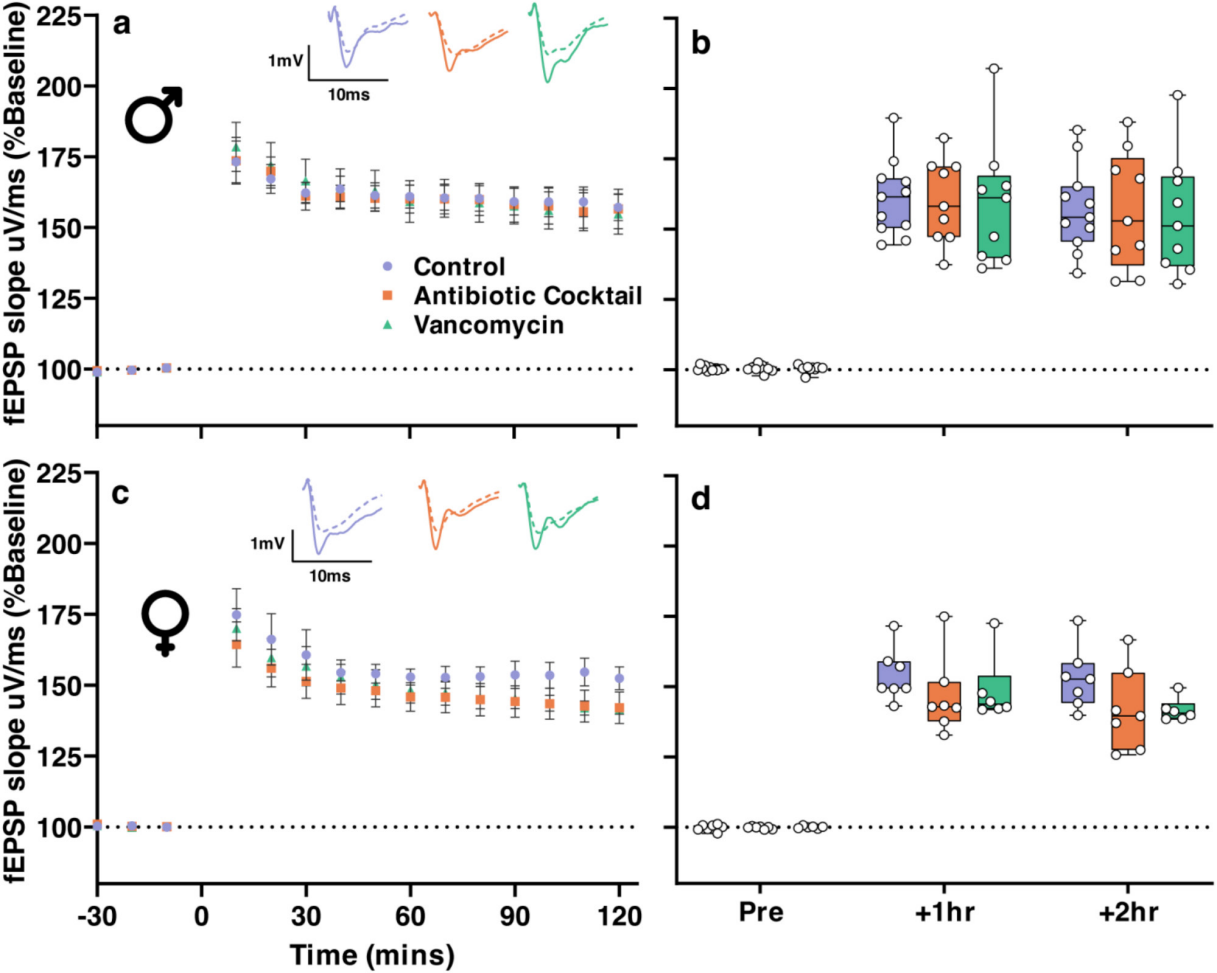
(a and c) Timeline of normalised CA1 fEPSP slopes for control, antibiotic cocktail and vancomycin‐treated groups with male slices featured in (a) and female slices featured in (c). No difference was noted between treatment groups. Representative traces at both pre (dashed lines) and post (solid lines) for each group. Scale bar is 1 mV × 10 ms. (b and d) Box and whisker chart summarising pre‐, 1 h‐post and 2 h‐post LTP induction fEPSP responses. Boxes represent the interquartile range with the median indicated, and whiskers represent the minimum and maximum values. Male *N* Control/Antibiotic Cocktail/Vancomycin = 11/9/9, Female N Control/Antibiotic Cocktail/Vancomycin = 7/7/6.

Post 30‐min baseline, LTP was induced using 3×TBS (Figure 2). This produced a rapid potentiation of fEPSPs that lasted for the 2‐h recording period (Control: Mean^male^ = 157.1% SEM 4.98, Mean^female^ = 152.9% SEM 6.2, Antibiotic Cocktail: Mean^male^ = 156.6% SEM 5.5, Mean^female^ = 141.9% SEM 6.2, Vancomycin: Mean^male^ = 154.8% SEM 5.5, Mean^female^ = 141.7% SEM 6.7). LTP did not change in ABX‐ nor Vancomycin‐treated groups when compared to controls. General Linear Model with Repeated Measures at 2‐h post‐TBS revealed no significant effect of ABX (*F*(2, 43) = 0.809, *p* = 0.452), but did reveal a significant effect of sex (*F*(1, 43) = 4.992, *p* = 0.031). No interaction between sex and antibiotic treatment was found (*F*(2, 43) = 0.487, *p* = 0.618).

### 3.3 Microbiome Depletion Does Not Affect Somatic Responses After Induction of Hippocampal LTP

**FIGURE 3:**
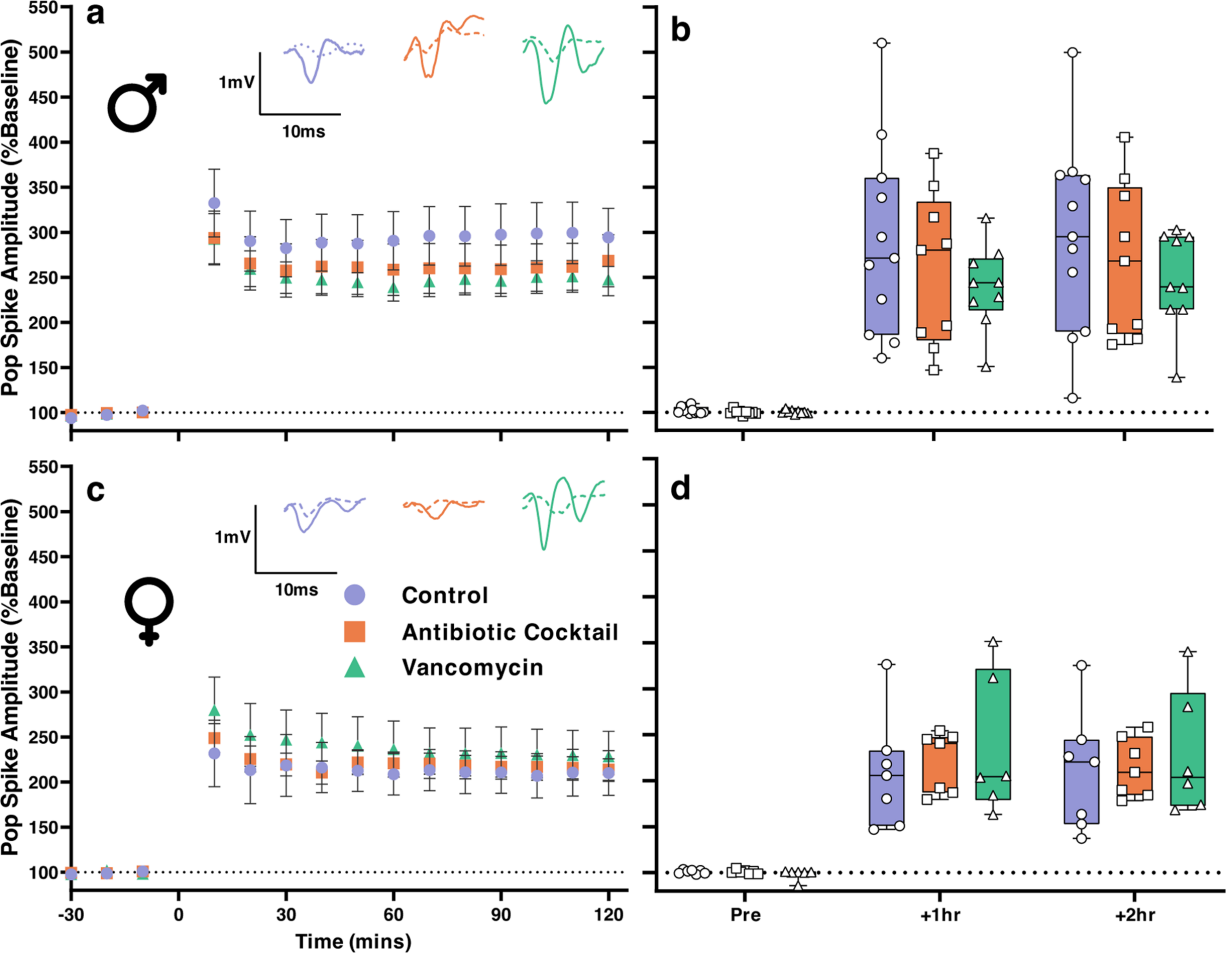
(a and c) Timeline of normalised CA1 pop. spike amplitudes for control, antibiotic cocktail‐ and vancomycin‐treated groups with male slices featured in (a) and female slices in (c). No difference was noted between treatment groups. Representative traces at both pre (dashed lines) and post (solid lines) for each group. Scale bar is 1 mV × 10 ms. (b and d) Box and whisker chart summarising pre‐, 1‐h post and 2‐h post LTP induction population spike responses. Boxes represent the interquartile range with the median indicated, and whiskers represent the minimum and maximum values. Male N Control/Antibiotic Cocktail/Vancomycin = 11/9/9, Female Control/Antibiotic Cocktail/Vancomycin = 7/7/6.

We concurrently recorded population spike responses to 3×TBS (Figure 3). In all groups, 3×TBS led to sustained potentiation of population spike amplitude at 2‐h post induction (Control: Mean^male^ = 294.5% SEM 23.1, Mean^female^ = 210.1% SEM 29.0, Antibiotic Cocktail: Mean^male^ = 268.5% SEM 25.6, Mean^female^ = 214.0% SEM 29.0, Vancomycin: Mean^male^ = 247.7% SEM 25.6, Mean^female^ = 228.5% SEM 31.3). General Linear Model with Repeated Measures at 2‐h post 3×TBS stimulation revealed no significant effect of microbiome depletion (*F*(2, 43) = 0.152, *p* = 0.860) and no interaction effect (*F*(2, 43) = 0.706, *p* = 0.499) but did reveal a significant effect of sex (*F*(2, 43) = 5.548, *p* = 0.023). Pairwise comparisons revealed the significant effect of sex only existed between control animals (*p* = 0.028). This shows that while population spike potentiation in response to 3×TBS is comparable between control and antibiotic‐treated adult mice, there may exist a subtle effect of microbiome treatment, which obfuscates sex differences otherwise present.

### 3.4 Microbiome Depletion Does Not Alter Excitatory Postsynaptic Potential‐Spike Coupling

**FIGURE 4:**
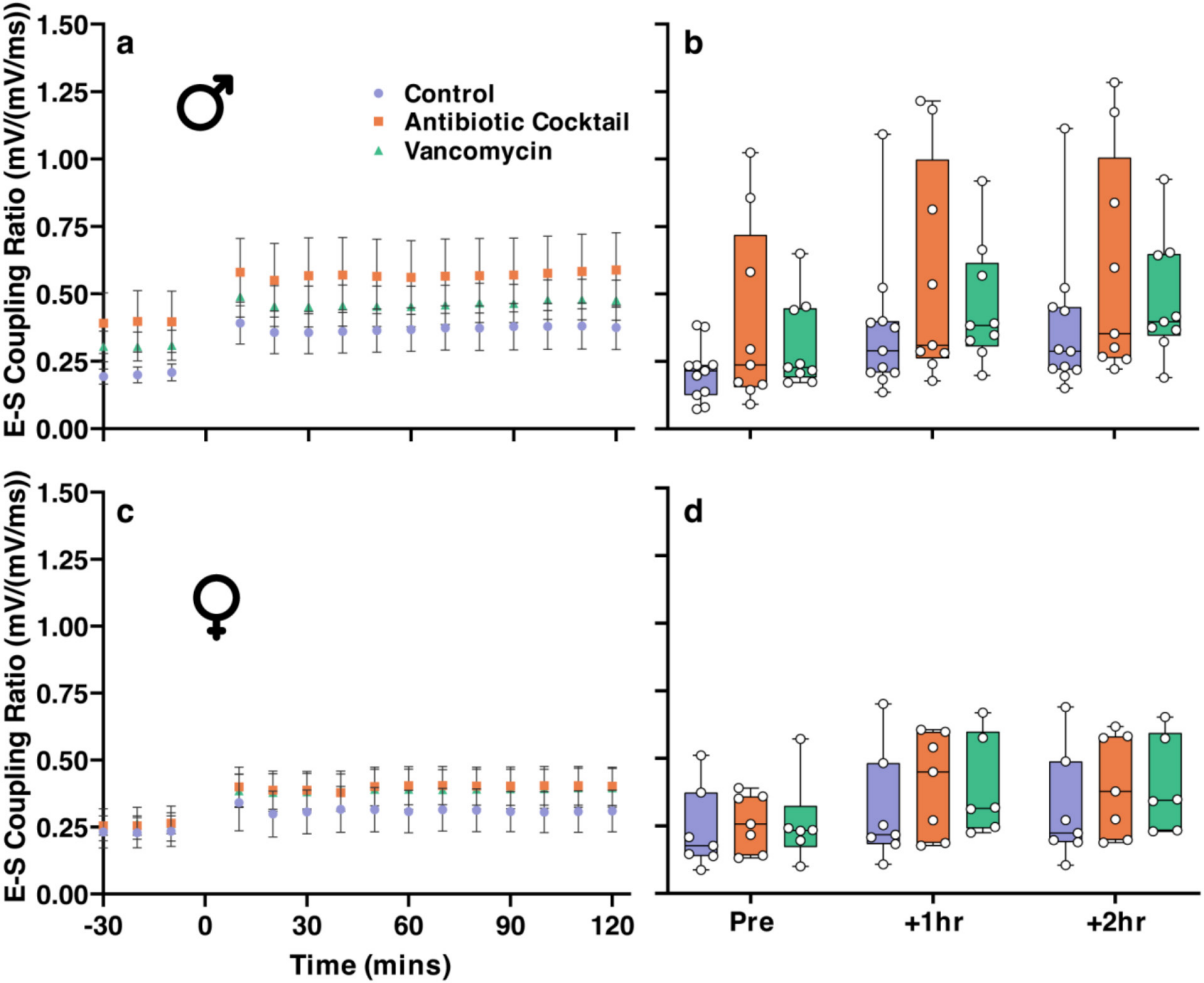
(a and c) Timeline of normalised CA1 E‐S coupling for control, antibiotic cocktail, and vancomycin‐treated groups with male slices featured in (a) and female slices in (c). No difference found between treatment groups (b) and (d). Box and whisker chart summarising pre‐, 1‐h post and 2‐h post LTP induction E‐S coupling. Boxes represent the interquartile range with the median indicated, and whiskers represent the minimum and maximum values. Male *N* Control/Antibiotic Cocktail/Vancomycin = 11/9/9, Female Control/Antibiotic Cocktail/Vancomycin = 7/7/6.

While not significant, the contrast between the effects of TBS on the dendritic and somatic responses of slices from control and antibiotic‐treated mice suggested a difference in the ability of the neuronal populations examined to integrate synaptic excitation to generate an action potential (E‐S coupling). Before 3 × TBS stimulation a general linear model with repeated measures revealed no significant effect of microbiome depletion (*F*(2, 43) = 1.415, *p* = 0.254), no significant effect of sex (*F*(1, 43) = 0.819, *p* = 0.370) and no interaction effect (*F*(2, 43) = 0.804, *p* = 0.454). All groups exhibited an increase in the E‐S coupling ratio (here representing an increase in population spike amplitude for a given fEPSP response) that is maintained for the duration of the recordings (Figure 4). However, the general linear model with repeated measures at 2‐h post 3×TBS stimulation also revealed no significant effect of microbiome depletion (*F*(2, 43) = 1.345, *p* = 0.271), no significant effect of sex (*F*(1, 43) = 1.976, *p* = 0.167) and no interaction effect (*F*(2, 43) = 0.238, *p* = 0.789).

## 4 Discussion

Previously, this lab found that GF upbringing produces hippocampal plasticity deficits affecting male but not female mice (Darch et al. 2021). Additionally, other studies show LTP behavioural correlates—learning and memory—can be disrupted by early adulthood microbiome depletion (Frohlich et al. 2016; Mohle et al. 2016). Further, similar antibiotic regimens in adult male mice have led to hippocampal dysfunction including reduced synaptic transmission and gamma oscillations (Çalışkan et al. 2022). Thus, we hypothesised hippocampal plasticity processes may be vulnerable to microbiome depletion during adulthood. In this study, we found that a 2‐week antibiotic treatment did not affect hippocampal LTP, short‐term plasticity as measured by PPF, or basal synaptic efficacy in male and female mice. These findings suggest these hippocampal properties are resilient to microbiome depletion in adulthood.

Given previously observed vulnerability of adulthood learning and memory to microbiome depletion, these findings were unexpected. Several factors may account for this discrepancy, including variations in microbiome depletion protocols, choices of learning and memory processes evaluated and potential sex differences. Previous data reported antibiotic treatment reduced memory retention, as assessed by NOR (Mohle et al. 2016). This study involved female mice exclusively, and a 7‐week antibiotic treatment, compared to the 2‐week treatment used here. Further, we have previously shown in male rats that treatment with an antibiotic cocktail including metronidazole in adulthood that continued throughout behavioural testing for a total of 13 weeks, led to spatial memory deficits in the Morris Water Maze (Hoban et al. 2016). This suggests a longer treatment duration may be necessary for functional hippocampal plasticity changes to manifest. The inclusion of metronidazole in earlier studies is worth noting. There is now a greater appreciation that this antibiotic readily penetrates the blood–brain barrier following oral administration (Hoban et al. 2016; Olson et al. 2005). This may induce local effects in the brain including neurotoxicity (Olson et al. 2005) through protein synthesis inhibition (Bradley et al. 1977), a process upon which LTP is dependent (Abraham and Williams 2008).

However, it is worth noting that some antibiotic depletion studies, which also avoid metronidazole, examined only adult male mice; differences were also found in NOR (Frohlich et al. 2016). While this test examines a hippocampal‐dependent behaviour (Broadbent et al. 2010), it also involves other regions such as the perirhinal cortex (Cinalli et al. 2020). Electrophysiological examination of this region may reveal changes to LTP. Further, conflicts exist regarding hippocampal involvement in object recognition memory (Barker and Warburton 2011; Cinalli et al. 2020; Cohen and Stackman 2015). Meanwhile, spatial memory, a process dependent on the dorsal hippocampus (Duda and Wesierska 2021), was insensitive to antibiotic administration (Frohlich et al. 2016). Our study, which examined the middorsal hippocampus, aligns with this.

Finally, other studies have shown that long‐term memory is affected in males by microbiome depletion utilising antibiotics starting at 6 weeks of age (Takahashi et al. 2024). However, this was measured by the passive avoidance test, which is once again more associated with the ventral hippocampus (Bryant and Barker 2020). In agreement with the lack of changes seen in synaptic plasticity in this study, they found spatial working memory as examined via the *y*‐maze test to be insensitive to antibiotic administration (Frohlich et al. 2016).

Taken together, there exists a clear bias in studies concerning microbiome depletion and learning and memory toward the examination of only a single sex. This may occlude the discovery of potential sex differences considering the previous microbiome‐associated sex differences noted by us (Darch et al. 2021) and others (Geary et al. 2021; Jaggar et al. 2020). Furthermore, longer microbiome depletion interventions and those starting at a younger age appear to have a greater capacity to induce changes in learning and memory processes (Frohlich et al. 2016; Mohle et al. 2016; Takahashi et al. 2024).

Although hippocampal plasticity has shown resilience to microbiome depletion in adulthood, in the current study, this does not preclude the possibility that microbiome depletion at earlier stages of development may affect this process. Evidence now points to learning and memory and synaptic plasticity being more vulnerable to insults during early neurodevelopment; previous work by this lab shows that maternal antibiotic administration can affect NOR in adult offspring (O'Connor et al. 2021) and that germ‐free adult mice display synaptic plasticity deficits (Darch et al. 2021). Early‐life microbiome interventions during various critical windows of neurodevelopment may then help paint a picture of learning and memory and synaptic plasticity being especially vulnerable during this period. Indeed, early‐life antibiotic administration has revealed gene expression changes in the amygdala and prefrontal cortex accompanied by enduring changes to anxiety‐like and compulsive behaviour (Lynch et al. 2023). Despite a prior study on early‐life antibiotic administration in rat pups reporting no spatial memory impairments that would be indicative of hippocampal plasticity deficits (O'Mahony et al. 2014), there is a large degree of variance in susceptibility to behavioural changes due to antibiotic administration (Olavarria‐Ramirez et al. 2023). Thus, the possibility of a critical window of plasticity development that is vulnerable to microbiome insult remains.

It is important to note that both early‐life and late‐life (ageing) may represent windows of heightened vulnerability to microbiota perturbations. Early neurodevelopment is characterised by increased brain plasticity and dynamic microbiota–brain signalling, making it more susceptible to disruption by antibiotic exposure, as suggested by both germ‐free and early‐life antibiotic studies (Liu et al. 2022; Lynch et al. 2024). Conversely, ageing is associated with natural declines in synaptic plasticity and alterations in microbiota composition, and emerging evidence indicates that microbiota disruption in older animals can exacerbate cognitive decline (Li et al. 2025; Li et al. 2020; Wu et al. 2021). While the present study focused on young adult mice, future investigations should examine these life stages to determine whether microbiota depletion exerts a greater impact on hippocampal function at these potentially sensitive periods.

Several limitations of the present study should be acknowledged. First, we did not perform 16S rRNA sequencing or total faecal DNA quantification immediately prior to culling in the electrophysiology cohort. While these measures would have provided direct confirmation of microbiota depletion, we used caecum enlargement, a well established anatomical marker of gut microbial depletion, as our primary proxy. Both groups that were treated with antibiotics showed marked increases in caecum weight, consistent with substantial microbiota reduction reported in prior work.

Second, only a single timepoint taken immediately after the 2‐week intervention was assessed. It is possible that this timing did not coincide with maximal microbial depletion, or that a longer or ongoing depletion period would be required to detect changes in hippocampal synaptic plasticity.

Third, our electrophysiological experiments were performed ex vivo. Removing the brain from the systemic milieu eliminates ongoing peripheral inputs, including microbiota‐derived metabolites and signalling molecules, which could be necessary to modulate synaptic plasticity in real time. In vivo recordings might therefore reveal effects of antibiotic treatment not captured in the present study.

Finally, we focused exclusively on hippocampal synaptic plasticity, specifically in the middorsal CA1 region. It remains possible that other hippocampal subregions, or entirely different brain areas (e.g., perirhinal cortex and ventral hippocampus), could show altered plasticity following microbiota depletion. These possibilities merit further investigation in future studies.

We conclude that microbiome depletion does not lead to any robust alteration of hippocampal LTP or electrophysiological measures in adulthood. Given that we have shown that antibiotic depletion in adolescence leads to more robust changes than that in adulthood, when examining behavioural and transcriptomic measures in adulthood (Lach et al. 2020), future work should target ages at which evidence suggests hippocampal plasticity may be more vulnerable.

## Author Contributions

**Michael K. Collins:** conceptualization, investigation and data curation (lead), formal analysis (equal), methodology (lead), visualization (lead), writing – original draft preparation and review and editing (lead). **Henry Darch:** conceptualization, investigation and data curation, formal analysis (equal), methodology. **Loreto Olavarría‐Ramírez:** investigation and data curation. **Cian McCafferty:** conceptualization, writing – review and editing. **Kenneth J. O'Riordan:** conceptualization, methodology, visualization, writing – original draft preparation and review and editing, supervision. **John F. Cryan:** conceptualization (lead), funding acquisition, methodology, writing – review and editing, supervision (lead).

## Funding

This work was supported by the H2020 Marie Skłodowska‐Curie Actions, Marie Sklodowska‐Curie (grant agreement No. 754535), Irish Research Council (IRC GOIPG/2021/942), and Science Foundation Ireland (SFI/12/RC/2273_P2).

## Ethics Statement

All experiments were conducted in accordance with the guidelines of European Directive 86/609/EEC and the Recommendation 2007/526/65/EC and were approved by the local Animal Care and Use Committees. All efforts were made to minimise animal suffering and to reduce the number of animals used.

## Conflicts of Interest

J.F.C. has received research funding from Cremo, Dupont/IFF, Nutricia and Pharmavite. He has also been an invited speaker at meetings organised by Alimentary Health, Alkermes, Ordesa and Yakult and has served as a consultant for Nestle. K.J.O. has received honoraria from Sanofi Genzyme and Danone. The content of this paper has neither been influenced nor constrained by this support. All other authors declare no conflicts of interest.

## Data Availability

The data that support the findings of this study are available from the corresponding author upon reasonable request.

## References

[ejn70346-bib-0001] Abraham, W. C. , and J. M. Williams . 2008. “LTP Maintenance and Its Protein Synthesis‐Dependence.” Neurobiology of Learning and Memory 89, no. 3: 260–268. 10.1016/j.nlm.2007.10.001. 17997332

[ejn70346-bib-0002] Alemohammad, S. M. A. , S. M. R. Noori , E. Samarbafzadeh , and S. M. A. Noori . 2022. “The Role of the Gut Microbiota and Nutrition on Spatial Learning and Spatial Memory: A Mini Review Based on Animal Studies.” Molecular Biology Reports 49, no. 2: 1551–1563. 10.1007/s11033-021-07078-2. 35028854

[ejn70346-bib-0003] Barker, G. R. , and E. C. Warburton . 2011. “When Is the Hippocampus Involved in Recognition Memory?” Journal of Neuroscience 31, no. 29: 10721–10731. 10.1523/JNEUROSCI.6413-10.2011. 21775615 PMC6622630

[ejn70346-bib-0004] Bercik, P. , E. Denou , J. Collins , et al. 2011. “The Intestinal Microbiota Affect Central Levels of Brain‐Derived Neurotropic Factor and Behavior in Mice.” Gastroenterology 141, no. 2: 599–609. 10.1053/j.gastro.2011.04.052. 21683077

[ejn70346-bib-0005] Bliss, T. V. , and T. Lomo . 1973. “Long‐Lasting Potentiation of Synaptic Transmission in the Dentate Area of the Anaesthetized Rabbit Following Stimulation of the Perforant Path.” Journal of Physiology 232, no. 2: 331–356. 10.1113/jphysiol.1973.sp010273. 4727084 PMC1350458

[ejn70346-bib-0006] Boehme, M. , K. E. Guzzetta , T. F. S. Bastiaanssen , et al. 2021. “Microbiota From Young Mice Counteracts Selective Age‐Associated Behavioral Deficits.” Nature Aging 1, no. 8: 666–676. 10.1038/s43587-021-00093-9. 37117767

[ejn70346-bib-0007] Bradley, W. G. , I. J. Karlsson , and C. G. Rassol . 1977. “Metronidazole Neuropathy.” British Medical Journal 2, no. 6087: 610–611. 10.1136/bmj.2.6087.610. 198056 PMC1631560

[ejn70346-bib-0008] Broadbent, N. J. , S. Gaskin , L. R. Squire , and R. E. Clark . 2010. “Object Recognition Memory and the Rodent Hippocampus.” Learning & Memory 17, no. 1: 5–11. 10.1101/lm.1650110. 20028732 PMC2807177

[ejn70346-bib-0009] Bryant, K. G. , and J. M. Barker . 2020. “Arbitration of Approach‐Avoidance Conflict by Ventral Hippocampus.” Frontiers in Neuroscience 14: 615337. 10.3389/fnins.2020.615337. 33390895 PMC7773818

[ejn70346-bib-0010] Çalışkan, G. , T. French , S. Enrile Lacalle , et al. 2022. “Antibiotic‐Induced Gut Dysbiosis Leads to Activation of Microglia and Impairment of Cholinergic Gamma Oscillations in the Hippocampus.” Brain, Behavior, and Immunity 99: 203–217. 10.1016/j.bbi.2021.10.007. 34673174

[ejn70346-bib-0011] Chandra, S. , S. S. Sisodia , and R. J. Vassar . 2023. “The Gut Microbiome in Alzheimer's Disease: What We Know and What Remains to Be Explored.” Molecular Neurodegeneration 18, no. 1: 9. 10.1186/s13024-023-00595-7. 36721148 PMC9889249

[ejn70346-bib-0012] Cinalli, D. A., Jr. , S. J. Cohen , K. Guthrie , and R. W. Stackman Jr. 2020. “Object Recognition Memory: Distinct yet Complementary Roles of the Mouse CA1 and Perirhinal Cortex.” Frontiers in Molecular Neuroscience 13: 527543. 10.3389/fnmol.2020.527543. 33192287 PMC7642692

[ejn70346-bib-0013] Cohen, S. J. , and R. W. Stackman Jr. 2015. “Assessing Rodent Hippocampal Involvement in the Novel Object Recognition Task. A Review.” Behavioural Brain Research 285: 105–117. 10.1016/j.bbr.2014.08.002. 25169255 PMC7008635

[ejn70346-bib-0014] Cordella, F. , C. Sanchini , M. Rosito , et al. 2021. “Antibiotics Treatment Modulates Microglia‐Synapses Interaction.” Cells 10, no. 10: 2648. 10.3390/cells10102648. 34685628 PMC8534187

[ejn70346-bib-0015] Creager, R. , T. Dunwiddie , and G. Lynch . 1980. “Paired‐Pulse and Frequency Facilitation in the CA1 Region of the In Vitro Rat Hippocampus.” Journal of Physiology 299: 409–424. 10.1113/jphysiol.1980.sp013133. 7381775 PMC1279233

[ejn70346-bib-0016] Cryan, J. F. , K. J. O'Riordan , C. S. M. Cowan , et al. 2019. “The Microbiota‐Gut‐Brain Axis.” Physiological Reviews 99, no. 4: 1877–2013. 10.1152/physrev.00018.2018. 31460832

[ejn70346-bib-0017] Darch, H. T. , M. K. Collins , K. J. O'Riordan , and J. F. Cryan . 2021. “Microbial Memories: Sex‐Dependent Impact of the Gut Microbiome on Hippocampal Plasticity.” European Journal of Neuroscience 54, no. 4: 5235–5244. 10.1111/ejn.15119. 33458858 PMC8451864

[ejn70346-bib-0018] Distrutti, E. , J. A. O'Reilly , C. McDonald , et al. 2014. “Modulation of Intestinal Microbiota by the Probiotic VSL#3 Resets Brain Gene Expression and Ameliorates the Age‐Related Deficit in LTP.” PLoS ONE 9, no. 9: e106503. 10.1371/journal.pone.0106503. 25202975 PMC4159266

[ejn70346-bib-0019] Duda, W. , and M. Wesierska . 2021. “Spatial Working Memory in Rats: Crucial Role of the Hippocampus in the Allothetic Place Avoidance Alternation Task Demanding Stimuli Segregation.” Behavioural Brain Research 412: 113414. 10.1016/j.bbr.2021.113414. 34119508

[ejn70346-bib-0020] Frohlich, E. E. , A. Farzi , R. Mayerhofer , et al. 2016. “Cognitive Impairment by Antibiotic‐Induced Gut Dysbiosis: Analysis of Gut Microbiota‐Brain Communication.” Brain, Behavior, and Immunity 56: 140–155. 10.1016/j.bbi.2016.02.020. 26923630 PMC5014122

[ejn70346-bib-0021] Geary, C. G. , V. C. Wilk , K. L. Barton , et al. 2021. “Sex Differences in Gut Microbiota Modulation of Aversive Conditioning, Open Field Activity, and Basolateral Amygdala Dendritic Spine Density.” Journal of Neuroscience Research 99, no. 7: 1780–1801. 10.1002/jnr.24848. 33951219

[ejn70346-bib-0022] Hoban, A. E. , R. D. Moloney , A. V. Golubeva , et al. 2016. “Behavioural and Neurochemical Consequences of Chronic Gut Microbiota Depletion During Adulthood in the Rat.” Neuroscience 339: 463–477. 10.1016/j.neuroscience.2016.10.003. 27742460

[ejn70346-bib-0023] Jackman, S. L. , and W. G. Regehr . 2017. “The Mechanisms and Functions of Synaptic Facilitation.” Neuron 94, no. 3: 447–464. 10.1016/j.neuron.2017.02.047. 28472650 PMC5865607

[ejn70346-bib-0024] Jaggar, M. , K. Rea , S. Spichak , T. G. Dinan , and J. F. Cryan . 2020. “You've Got Male: Sex and the Microbiota‐Gut‐Brain Axis Across the Lifespan.” Frontiers in Neuroendocrinology 56: 100815. 10.1016/j.yfrne.2019.100815. 31805290

[ejn70346-bib-0025] Kennedy, M. B. 2013. “Synaptic Signaling in Learning and Memory.” Cold Spring Harbor Perspectives in Biology 8, no. 2: a016824. 10.1101/cshperspect.a016824. 24379319 PMC4743082

[ejn70346-bib-0026] Kim, C. H. , K. Takamiya , R. S. Petralia , et al. 2005. “Persistent Hippocampal CA1 LTP in Mice Lacking the C‐Terminal PDZ Ligand of GluR1.” Nature Neuroscience 8, no. 8: 985–987. 10.1038/nn1432. 16007085

[ejn70346-bib-0027] Kuijer, E. J. , and L. Steenbergen . 2023. “The Microbiota‐Gut‐Brain Axis in Hippocampus‐Dependent Learning and Memory: Current State and Future Challenges.” Neuroscience and Biobehavioral Reviews 152: 105296. 10.1016/j.neubiorev.2023.105296. 37380040

[ejn70346-bib-0028] Lach, G. , C. Fulling , T. F. S. Bastiaanssen , et al. 2020. “Enduring Neurobehavioral Effects Induced by Microbiota Depletion During the Adolescent Period.” Translational Psychiatry 10, no. 1: 382. 10.1038/s41398-020-01073-0. 33159036 PMC7648059

[ejn70346-bib-0029] Li, M. , J. Ren , Y. Bao , et al. 2025. “Aged Gut Microbiota Contributes to Cognitive Impairment and Hippocampal Synapse Loss in Mice.” Aging Cell 24, no. 7: e70064. 10.1111/acel.70064. 40219707 PMC12266779

[ejn70346-bib-0030] Li, Y. , L. Ning , Y. Yin , et al. 2020. “Age‐Related Shifts in Gut Microbiota Contribute to Cognitive Decline in Aged Rats.” Aging (Albany NY) 12, no. 9: 7801–7817. 10.18632/aging.103093. 32357144 PMC7244050

[ejn70346-bib-0031] Liu, G. , Q. Yu , B. Tan , et al. 2022. “Gut Dysbiosis Impairs Hippocampal Plasticity and Behaviors by Remodeling Serum Metabolome.” Gut Microbes 14, no. 1: 2104089. 10.1080/19490976.2022.2104089. 35876011 PMC9327780

[ejn70346-bib-0032] Lu, B. , G. Nagappan , and Y. Lu . 2014. “BDNF and Synaptic Plasticity, Cognitive Function, and Dysfunction.” Handbook of Experimental Pharmacology 220: 223–250. 10.1007/978-3-642-45106-5_9. 24668475

[ejn70346-bib-0033] Lynch, C. M. K. , C. S. M. Cowan , T. F. S. Bastiaanssen , et al. 2023. “Critical Windows of Early‐Life Microbiota Disruption on Behaviour, Neuroimmune Function, and Neurodevelopment.” Brain, Behavior, and Immunity 108: 309–327. 10.1016/j.bbi.2022.12.008. 36535610

[ejn70346-bib-0034] Lynch, C. M. K. , J. Nagpal , P. Luczynski , et al. 2024. “Germ‐Free Animals.” In The Gut‐Brain Axis, edited by N. Hyland and C. Stanton , 401–454. Academic Press. 10.1016/b978-0-323-99971-7.00012-6.

[ejn70346-bib-0035] Menigoz, A. , T. Ahmed , V. Sabanov , et al. 2016. “TRPM4‐Dependent Post‐Synaptic Depolarization Is Essential for the Induction of NMDA Receptor‐Dependent LTP in CA1 Hippocampal Neurons.” Pflügers Archiv 468, no. 4: 593–607. 10.1007/s00424-015-1764-7. 26631168 PMC4792339

[ejn70346-bib-0036] Mohle, L. , D. Mattei , M. M. Heimesaat , et al. 2016. “Ly6C(Hi) Monocytes Provide a Link Between Antibiotic‐Induced Changes in Gut Microbiota and Adult Hippocampal Neurogenesis.” Cell Reports 15, no. 9: 1945–1956. 10.1016/j.celrep.2016.04.074. 27210745

[ejn70346-bib-0037] Neufeld, K. M. , N. Kang , J. Bienenstock , and J. A. Foster . 2011. “Reduced Anxiety‐Like Behavior and Central Neurochemical Change in Germ‐Free Mice.” Neurogastroenterology and Motility 23, no. 3: 255–264. 10.1111/j.1365-2982.2010.01620.x. 21054680

[ejn70346-bib-0038] O'Connor, R. , G. M. Moloney , C. Fulling , et al. 2021. “Maternal Antibiotic Administration During a Critical Developmental Window Has Enduring Neurobehavioural Effects in Offspring Mice [Article].” Behavioural Brain Research 404: 113156. 10.1016/j.bbr.2021.113156. 33571573

[ejn70346-bib-0039] O'Dell, T. J. , E. R. Kandel , and S. G. Grant . 1991. “Long‐Term Potentiation in the Hippocampus Is Blocked by Tyrosine Kinase Inhibitors.” Nature 353, no. 6344: 558–560. 10.1038/353558a0. 1656271

[ejn70346-bib-0040] Olavarria‐Ramirez, L. , J. Cooney‐Quane , G. Murphy , C. P. McCafferty , J. F. Cryan , and S. Dockray . 2023. “A Systematic Review of the Effects of Gut Microbiota Depletion on Social and Anxiety‐Related Behaviours in Adult Rodents: Implications for Translational Research.” Neuroscience and Biobehavioral Reviews 145: 105013. 10.1016/j.neubiorev.2022.105013. 36566805

[ejn70346-bib-0041] Olson, E. J. , S. C. Morales , A. S. McVey , and D. W. Hayden . 2005. “Putative Metronidazole Neurotoxicosis in a Cat.” Veterinary Pathology 42, no. 5: 665–669. 10.1354/vp.42-5-665. 16145214

[ejn70346-bib-0042] O'Mahony, S. M. , V. D. Felice , K. Nally , et al. 2014. “Disturbance of the Gut Microbiota in Early‐Life Selectively Affects Visceral Pain in Adulthood Without Impacting Cognitive or Anxiety‐Related Behaviors in Male Rats.” Neuroscience 277: 885–901. 10.1016/j.neuroscience.2014.07.054. 25088912

[ejn70346-bib-0043] Papouin, T. , and P. G. Haydon . 2018. “Obtaining Acute Brain Slices.” Bio‐Protocol 8, no. 2: e2699. 10.21769/BioProtoc.2699. 29552595 PMC5856250

[ejn70346-bib-0044] Renson, A. , L. J. Kasselman , J. B. Dowd , L. Waldron , H. E. Jones , and P. Herd . 2020. “Gut Bacterial Taxonomic Abundances Vary With Cognition, Personality, and Mood in the Wisconsin Longitudinal Study.” Brain Behav Immun Health 9: 100155. 10.1016/j.bbih.2020.100155. 34589897 PMC8474555

[ejn70346-bib-0045] Shaban, H. , R. O'Connor , S. V. Ovsepian , T. G. Dinan , J. F. Cryan , and H. Schellekens . 2017. “Electrophysiological Approaches to Unravel the Neurobiological Basis of Appetite and Satiety: Use of the Multielectrode Array as a Screening Strategy.” Drug Discovery Today 22, no. 1: 31–42. 10.1016/j.drudis.2016.09.003. 27634341

[ejn70346-bib-0046] Takahashi, K. , K. Kurokawa , K. Miyagawa , A. Mochida‐Saito , H. Takeda , and M. Tsuji . 2024. “Repeated Antibiotic Drug Treatment Negatively Affects Memory Function and Glutamatergic Nervous System of the Hippocampus in Mice.” Neuroscience Letters 825: 137711. 10.1016/j.neulet.2024.137711. 38432356

[ejn70346-bib-0047] Woo, N. H. , H. K. Teng , C. J. Siao , et al. 2005. “Activation of p75NTR by proBDNF Facilitates Hippocampal Long‐Term Depression.” Nature Neuroscience 8, no. 8: 1069–1077. 10.1038/nn1510. 16025106

[ejn70346-bib-0048] Wu, M.‐L. , X.‐Q. Yang , L. Xue , W. Duan , and J.‐R. Du . 2021. “Age‐Related Cognitive Decline Is Associated With Microbiota‐Gut‐Brain Axis Disorders and Neuroinflammation in Mice.” Behavioural Brain Research 402: 113125. 10.1016/j.bbr.2021.113125. 33422597

